# Effect of ERAS-guided refined nursing on early mobilization after lung cancer surgery: a retrospective cohort study

**DOI:** 10.3389/fsurg.2026.1767707

**Published:** 2026-04-01

**Authors:** Yan Wang, Bin Wang, Fang Qi, Xiangnan Li, Jing Zhang

**Affiliations:** Department of Thoracic Surgery, Tangshan People’s Hospital, Tangshan, Hebei, China,

**Keywords:** early mobilization, enhanced recovery after surgery, lung cancer, postoperative recovery, refined nursing, thoracic surgery

## Abstract

**Background:**

Early-stage mobilization is a major component of Enhanced Recovery After Surgery (ERAS) for lung cancer surgery.

**Objective:**

To investigate the impact of ERAS-based refined nursing on early postoperative activities and recovery.

**Methods:**

This was a retrospective cohort study of 136 patients who received a radical lung cancer operation, with 68 patients assigned to the conventional nursing group and 68 to the ERAS-guided refined nursing group. Early mobilization parameters were time to first ambulation, ambulation distance at 24 h and 48 h, Timed Up and Go (TUG) test, and Barthel Index. Secondary outcomes were recovery parameters, pain, complications, nursing compliance, and patient satisfaction.

**Results:**

Patients in the ERAS group demonstrated significantly earlier ambulation, higher early walking distances, better functional exercise capacity and Barthel Index score than those in the conventional group (*P* < 0.001). The postoperative recovery was enhanced with earlier gastrointestinal function, reduced duration of drainage and catheterization, and shorter length of stay (LOS, *P* < 0.001). Significant reductions in postoperative pain and usage of analgesic pump were observed. The incidence of overall postoperative complications was decreased in the ERAS group, especially the pulmonary infection, deep venous thrombosis (DVT) and arrhythmia. Nursing compliance and patient satisfaction were also significantly higher in the ERAS group.

**Conclusion:**

ERAS-based refined nursing was significantly associated with earlier mobilization, faster postoperative recovery, a lower incidence of complications, and improved nursing compliance and patient satisfaction after lung cancer surgery.

## 1 Introduction

Lung cancer remains a leading cause of cancer-related mortality worldwide, and surgical resection is the cornerstone of curative treatment for early-stage disease and selected locally advanced cases. With advances in operative techniques and perioperative care, attention has increasingly shifted from survival alone to functional recovery and postoperative quality of life. Patients undergoing thoracotomy or video-assisted thoracoscopic surgery (VATS) are at risk of postoperative pulmonary and thromboembolic complications, muscle deconditioning, and prolonged hospitalization, particularly when bed rest is extended and ambulation is delayed (1, 2). These adverse outcomes impose substantial patient, caregiver, and financial burden, highlighting the need for systematic approaches to facilitate early mobilization and recovery.

Enhanced recovery after surgery (ERAS) is an evidence-based, multidisciplinary perioperative pathway designed to attenuate surgical stress, preserve physiological function, and promote early return to normal activities. Following its initial adoption in colorectal surgery, ERAS principles have been adapted across multiple surgical specialties (3, 4). Key components commonly include preoperative counselling, optimization of nutrition and comorbidities, minimally invasive techniques when feasible, multimodal analgesia, early oral nutrition, and early mobilization. In thoracic surgery, ERAS programs aim to reduce postoperative pulmonary complications, chest drainage duration, and length of hospital stay (LOS) (2).

Accumulating evidence supports ERAS implementation in lung resection. Systematic reviews and meta-analyses have shown that thoracic ERAS can reduce LOS by approximately 2–3 days and lower overall complication rates without increasing readmissions or mortality (1, 5). Studies in lung cancer populations further suggest associations with fewer pulmonary and cardiac complications, reduced hospital costs, and earlier initiation or completion of adjuvant chemotherapy in non-small cell lung cancer (1, 6, 7). In response to this evidence base, the European Society of Thoracic Surgeons (ESTS) issued comprehensive guidelines for enhanced recovery after lung surgery in 2019, presenting 45 evidence-based recommendations and explicitly emphasizing early mobilization as a fundamental element (8).

Early postoperative mobilization is now widely regarded as a cornerstone of ERAS. Prolonged inactivity after major surgery contributes to rapid muscle atrophy, impaired ventilatory function, orthostatic intolerance, and increased risk of venous thromboembolism (9, 10). Across ERAS literature, earlier mobilization is consistently associated with fewer complications and shorter LOS (9). In lung resection, prospective observational studies have reported that ambulation on the day of surgery or within the first 24 h can reduce morbidity, opioid use, chest tube duration, and LOS compared with later ambulation (11, 12). However, mobilization targets and practical nursing strategies to achieve them remain insufficiently standardized (10, 13).

Despite these advances, implementation of early mobilization within thoracic ERAS remains variable, and nursing-directed approaches are often not clearly specified. Systematic reviews have highlighted substantial heterogeneity in ERAS protocols after lung resection, as well as variability in compliance and in the monitoring of functional outcomes (2, 14). Moreover, early mobilization is frequently embedded within multi-component pathways and inferred indirectly through composite outcomes such as LOS or overall complication rates rather than being evaluated using dedicated quantitative mobilization endpoints (1, 15). Surveys of healthcare practitioners involved in thoracic ERAS programs also indicate gaps in knowledge, attitudes, and practices related to key elements such as early ambulation, including at high-volume tertiary centers (16, 17). Collectively, these findings suggest that pragmatic, reproducible nursing interventions are needed to translate ERAS principles into measurable gains in early postoperative mobility.

Refined nursing (also termed precision or individualized nursing) is a structured, patient-centered approach that integrates detailed assessment, stratified risk management, and tailored perioperative interventions. Within an ERAS framework, refined nursing may incorporate standardized education, psychological support, respiratory and physical training, optimized pain management, and stepwise mobilization plans aligned with patient status. Emerging evidence in thoracic surgery suggests that ERAS-based refined nursing can improve physiological indices, enhance quality of life, and support postoperative recovery compared with conventional nursing models. For example, a recent study in patients undergoing radical lung cancer surgery reported that ERAS-based refined nursing improved clinical and physiological indices in the early postoperative period (18). Another prospective study found that refined nursing facilitated recovery, improved respiratory function, and enhanced overall well-being after thoracic procedures (19). Nurse-driven ERAS programs in older thoracic patients further indicate that protocolized nursing leadership can strengthen pathway adherence and functional outcomes (20).

Nevertheless, few studies have evaluated ERAS-guided refined nursing with early mobilization as a primary endpoint in lung cancer surgery. Most thoracic ERAS reports emphasize LOS, complication rates, and costs, while early ambulation is often treated as a process indicator rather than a clinically meaningful outcome (1, 15, 21). Quantitative measures such as time to first ambulation, early walking distance, simple functional performance tests, and early activities of daily living scores may more directly reflect functional recovery and nursing effectiveness than traditional metrics alone, yet these endpoints have not been consistently integrated into ERAS-oriented nursing research in lung cancer.

Therefore, this study aimed to investigate the effect of ERAS-guided refined nursing on early postoperative mobilization and related recovery outcomes in patients undergoing lung cancer surgery. By clarifying the contribution of refined nursing to early functional recovery, our findings may inform the development and broader implementation of nursing-driven ERAS protocols in thoracic oncology practice.

## 2 Materials and methods

### 2.1 Study design and participants

This study is a retrospective, comparative cohort study conducted in the Department of Thoracic Surgery, Tangshan People's Hospital. A total of 136 consecutive patients who underwent elective radical surgery for lung cancer between January 2025 and July 2025 were included. ERAS-guided refined nursing was implemented in our ward starting on April 30, 2025. Accordingly, patients who underwent surgery before May 1, 2025 received conventional nursing care (conventional group, *n* = 68), whereas those who underwent surgery on or after May 1, 2025 received ERAS-guided refined nursing (ERAS group, *n* = 68). To minimize contamination, the nursing pathway was standardized and delivered according to the corresponding protocol during each period.

Nevertheless, as this was a retrospective cohort study, *a priori* sample size calculation was not performed. All consecutive eligible patients during the study period were included, and a *post hoc* power analysis based on the primary outcome demonstrated that the final sample size provided adequate statistical power (power > 0.80).

This study conformed to the principles outlined in the Declaration of Helsinki, and the protocol was approved by the Institutional Ethics Committee of the Tangshan People's Hospital. The need for written informed consent was waived due to the study's retrospective design.

### 2.2 Inclusion and exclusion criteria

#### 2.2.1 Inclusion criteria

The patients were included in this study if they met the following criteria: be aged 18 years or older, have a pathologically confirmed diagnosis of primary lung cancer, and be scheduled to receive elective radical surgery for lung cancer. Furthermore, only patients with an American Society of Anesthesiologists (ASA) physical status classification of I-III were included. Full clinical, perioperative and nursing records were necessary for all the participants.

#### 2.2.2 Exclusion criteria

Patients were excluded if they had emergency surgery or if planned procedure was converted to palliative procedure. People with an antecedent of severe cardiopulmonary dysfunction, coagulation disorder or psychiatric disease which may affect compliance to perioperative care were also excluded. Besides, patients who had preoperative distant metastasis or who had inadequate clinical information were also excluded from this study.

### 2.3 Nursing interventions

#### 2.3.1 Conventional nursing group

Patients in the conventional group received routine perioperative nursing care during the perioperative period: the patients received preoperative counseling and health education, regular fasting and enema, monitoring of vital signs after surgery, conventional management of analgesics, general respiratory nursing, and routine guidance on the postoperative activities according to the hospital norms.

#### 2.3.2 ERAS-guided refined nursing during preoperative phase

(1) Education and counseling
  A structured session conducted at least once daily, covering the surgical procedure, pain management strategies, mobilization goals, and discharge preparation. These efforts were delivered by trained thoracic nurses using a checklist; documented in nursing records.
(2) Respiratory function training
  Daily training consisting of deep breathing, effective coughing exercises, and incentive spirometry, which were initiated 2 days before surgery, with 3–4 sessions/day, 10–15 min each. This training was supervised and reinforced by bedside nurses.
(3) Smoking cessation counseling
  When applicable, provided and documented in nursing records.
(4) Nutritional assessment and guidance
  Patients were assessed for nutritional risk using the NRS-2002 scale, and those identified as at risk were provided with nutritional support.
(5) Psychological support
  Perioperative anxiety reduction strategies such as the relaxation techniques to enhance patient comfort and sleep, particularly for those with high preoperative anxiety.

#### 2.3.3 ERAS-guided refined nursing during intraoperative cooperation

(1) Normothermia management
  Active warming measures such as the warming blankets and warmed intravenous fluids were employed during surgery to prevent hypothermia, thus to provide a core temperature of ≥36 °C for the patients.
(2) Fluid management documentation
  Nurses assisted in documenting fluid input and output, collaborating closely with the anesthesia team to ensure balanced perioperative fluid management.

#### 2.3.4 ERAS-guided refined nursing during postoperative phase

(1) Early mobilization protocol
  Patients were encouraged to sit up and begin ambulation as early as possible, with a goal of walking within 24 h of surgery if stable. Walking distance was progressively increased each day with nurse-assisted sessions. By POD 2, the goal was to achieve a walking distance of ≥150 meters.
(2) Pain management
  VAS scores were assessed 3 times daily from POD 1–3, with multimodal analgesia adjusted according to the patient's pain level. Pain management was provided with a combination of oral analgesics and PCA (patient-controlled analgesia) when needed.
(3) Respiratory exercises
  Deep breathing, coughing, and sputum clearance were practiced with a target of 3–4 sessions daily.
(4) Early oral intake
  Patients were encouraged to start with clear liquids on POD 1, progressing to soft foods by POD 2, based on tolerance. Nurses documented tolerance and any signs of nausea or vomiting.
(5) Catheter and drain removal
  The urinary catheter and chest drains were removed as early as possible when clinically appropriate, based on the physician's order and protocol for each case.
(6) Discharge preparation
  Nurses provided education on self-care after discharge, including wound care, mobilization exercises, pain management, and follow-up appointments.

### 2.4 Data collection

The clinical information was obtained from the electronic medical records of the hospital. Baseline characteristics were age, sex, body mass index (BMI), history of smoking, comorbidities, ASA classification, tumor stage, surgical procedure, operative duration, and intraoperative bleeding. More specifically, comorbidities such as hypertension, diabetes mellitus, and chronic obstructive pulmonary disease were thoroughly analyzed in this research.

### 2.5 Outcome measures

#### 2.5.1 Early mobilization outcomes

Several time related metrics of early mobilization were used such as: time to first ambulation after surgery, walking distance at 24 and 48 h postoperatively, and attainment of independent ambulation at 72 h. Functional mobility was also assessed by the Timed Up and Go (TUG) test on postoperative day 3. Moreover, activities of daily living were evaluated by the Barthel index on postoperative day 3 as a marker of early functional recovery.

#### 2.5.2 Definitions and assessment procedures

Time to first ambulation was defined as the elapsed time from the end of surgery to the first out-of-bed ambulation documented in the nursing record. Walking distance at 24 h and 48 h was measured by the bedside nurse using the ward's standardized walking-route measurement and recorded in the nursing chart. The TUG test was performed on postoperative day 3 following a standardized protocol: standing up from a chair, walking 3 meters, turning, returning, and sitting down; time recorded in seconds. Activities of daily living were assessed on postoperative day 3 using the Barthel Index, with higher scores indicating better function.

#### 2.5.3 Postoperative recovery indicators

Postoperative recovery was evaluated based on multiple clinical indicators, including the time to first flatus, duration of chest tube indwelling, duration of urinary catheterization, time to first oral intake, and length of postoperative hospital stay. These parameters were used to comprehensively reflect the speed and quality of postoperative recovery.

#### 2.5.4 Operational definitions and units

Time to first flatus was defined as the elapsed time (hours) from the end of surgery to the first patient-reported passage of flatus documented by nurses in the nursing and medical records. Time to first oral intake also referred to as the start of feed, was defined as the elapsed time (hours) from the end of surgery to the first tolerated oral intake documented in the nursing record. Duration of chest tube drainage, duration of urinary catheterization, and postoperative length of stay were recorded in days based on electronic medical records.

#### 2.5.5 Postoperative pain and comfort

Pain intensity was evaluated by visual analogue scale (VAS) during the first 3 days postoperatively. Other comfort-related outcomes were analgesic pump usage and postoperative nausea and vomiting (PONV).

VAS was assessed at fixed time points each day from postoperative day 1 to day 3 by trained nurses and recorded in the nursing chart, and PONV was defined as any documented nausea or vomiting episode within 72 h after surgery in nursing or medical records.

#### 2.5.6 Postoperative complications

Postoperative complications included pulmonary infection, atelectasis, arrhythmia, DVT, incision infection, reintubation, and intensive care unit admission (ICU) readmission. The presence of any complication and the total number of complications per patient were also recorded. Postoperative complications were identified from physician documentation, laboratory results, as well as nursing records during hospitalization.

#### 2.5.7 Nursing compliance and satisfaction

Compliance with nursing care was assessed based on early ambulation compliance, respiratory training accomplishment, and sputum expectoration. Nursing satisfaction was assessed with a 5-point Likert scale. Nursing compliance items were assessed using ward checklists and nursing documentation; satisfaction was collected at discharge using a 5-point Likert questionnaire.

### 2.6 Statistical analysis

Data analysis was performed with IBM SPSS (version 27.0, IBM Corp, Armonk, NY, USA). Continuous variables are presented as mean ± SD and compared using independent-samples *t*-tests. Categorical variables are presented as *n* (%) and compared using the *χ*^2^ test. When comparing multiple continuous variables, the Bonferroni correction was used to adjust the level of significance to control the risk of type I error. Unless otherwise specified, a two-sided *P* value < 0.05 was considered statistically significant. Bonferroni correction (adjusted *α* = 0.0125) was applied only to the continuous variables at baseline: age, BMI, operation time, and intraoperative blood loss.

## 3 Results

### 3.1 Baseline characteristics

In this research (Table 1) , a total of 136 patients were included in the analysis, with 68 patients in each group. Baseline demographic, clinical, tumor-stage, surgical, and perioperative characteristics were comparable between groups. There were no statistically significant differences in baseline characteristics, including age, sex, smoking history, comorbidities, ASA classification, tumor stage, surgical approach, operative time and intraoperative blood loss, between the two groups. BMI showed a nominal difference (*P* = 0.042), but did not reach the Bonferroni-adjusted threshold (*α* = 0.0125).

**Table 1:** Baseline demographic and clinical characteristics of the patients.

Variable	Conventional group	ERAS group	Statistics
	(*n* = 68)	(*n* = 68)	
Age, years (mean ± SD)	62.0 ± 8.0	61.0 ± 7.1	*P* = 0.476^a^
Sex, *n* (%)			
Male	40 (58.8%)	34 (50.0%)	*P* = 0.389
Female	28 (41.2%)	34 (50.0%)	
BMI, kg/m^2^ (mean ± SD)	23.3 ± 2.4	24.2 ± 2.8	*P* = 0.042^a^
Smoking history, *n* (%)			
No	23 (33.8%)	24 (35.3%)	*χ*^2^ = 0.033 *P* = 0.857
Yes	45 (66.2%)	44 (64.7%)	
Comorbidities, *n* (%)			
None	32 (47.1%)	32 (47.1%)	χ^2^ = 2.896 *P* = 0.408
Hypertension	20 (29.4%)	25 (36.8%)	
Diabetes mellitus	11 (16.2%)	5 (7.4%)	
COPD	5 (7.4%)	6 (8.8%)	
ASA classification, *n* (%)			
ASA I	6 (8.8%)	11 (16.2%)	χ^2^ = 2.661 *P* = 0.264
ASA II	49 (72.1%)	49 (72.1%)	
ASA III	13 (19.1%)	8 (11.8%)	
Tumor stage, *n* (%)			
Stage I	38 (55.9%)	32 (47.1%)	χ^2^ = 4.760 *P* = 0.093
Stage II	22 (32.4%)	18 (26.5%)	
Stage III	8 (11.8%)	18 (26.5%)	
Surgical approach, *n* (%)			
VATS	48 (70.6%)	47 (69.1%)	χ^2^ = 0.035 *P* = 0.852
Thoracotomy	20 (29.4%)	21 (30.9%)	
Operation time, min (mean ± SD)	161.1 ± 37.7	170.2 ± 33.8	*P* = 0.144^a^
Intraoperative blood loss, mL (mean ± SD)	209.7 ± 139.5	208.0 ± 126.9	*P* = 0.940^a^

^a^ Bonferroni-adjusted significance level of 0.0125 (*α* = 0.05/4 = 0.0125) should be applied to the marked variables: age, BMI, operation time and intraoperative blood loss.

### 3.2 Early mobilization outcomes

Patients in the ERAS group achieved earlier ambulation than those in the conventional group (20.7 ± 5.4 h vs. 27.4 ± 6.8 h, *P* < 0.001). Walking distance was significantly longer in the ERAS group at both 24 h (83.1 ± 25.0 m vs. 37.4 ± 18.5 m, *P* < 0.001) and 48 h postoperatively (182.3 ± 60.7 m vs. 117.4 ± 41.9 m, *P* < 0.001). Functional mobility on postoperative day 3 was also better in the ERAS group, as indicated by shorter TUG time (17.6 ± 3.9 s vs. 21.8 ± 5.0 s, *P* < 0.001) and higher Barthel Index score (78.5 ± 8.8 vs. 71.0 ± 9.6, *P* < 0.001) (Table 2).

**Table 2:** Comparison of early mobilization outcomes between the two groups.

Variable	Conventional group (*n* = 68)	ERAS group (*n* = 68)	t value	*P* value
Time to first ambulation, h (mean ± SD)	27.4 ± 6.8	20.7 ± 5.4	−6.291	<0.001
Walking distance at 24 h, m (mean ± SD)	37.4 ± 18.5	83.1 ± 25.0	12.108	<0.001
Walking distance at 48 h, m (mean ± SD)	117.4 ± 41.9	182.3 ± 60.7	7.258	<0.001
TUG test, s (postoperative day 3, mean ± SD)	21.8 ± 5.0	17.6 ± 3.9	−5.491	<0.001
Barthel index, postoperative day 3 (mean ± SD)	71.0 ± 9.6	78.5 ± 8.8	4.760	<0.001

### 3.3 Postoperative recovery indicators

Postoperative recovery was faster in the ERAS group. Gastrointestinal function recovered earlier, with shorter time to first flatus (37.8 ± 7.3 h vs. 56.1 ± 8.8 h, *P* < 0.001) and time to first oral intake (21.3 ± 5.2 h vs. 39.7 ± 8.2 h, *P* < 0.001). The ERAS group also had shorter duration of chest tube drainage (3.9 ± 1.2 d vs. 5.4 ± 1.3 d, *P* < 0.001) and urinary catheterization (2.2 ± 0.7 d vs. 3.8 ± 1.1 d, *P* < 0.001), and a reduced postoperative length of stay (7.2 ± 1.5 d vs. 9.4 ± 2.1 d, *P* < 0.001) (Table 3).

**Table 3:** Comparison of postoperative recovery indicators between the two groups.

Variable	Conventional group (*n* = 68)	ERAS group (*n* = 68)	t value	*P* value
Time to first flatus, h (mean ± SD)	56.1 ± 8.8	37.8 ± 7.3	−13.208	<0.001
Duration of chest tube drainage, d (mean ± SD)	5.4 ± 1.3	3.9 ± 1.2	−6.899	<0.001
Duration of urinary catheterization, d (mean ± SD)	3.8 ± 1.1	2.2 ± 0.7	−10.137	<0.001
Time to first oral intake, h (mean ± SD)	39.7 ± 8.2	21.3 ± 5.2	−15.660	<0.001
Length of postoperative hospital stay, d (mean ± SD)	9.4 ± 2.1	7.2 ± 1.5	−6.816	<0.001

h, hours; d, days. Time to first flatus and time to first oral intake were recorded in hours; chest tube duration, urinary catheter duration, and postoperative length of stay were recorded in days.

### 3.4 Pain and comfort

Postoperative pain was lower in the ERAS group across the first three postoperative days (4.6 ± 1.1 vs. 5.8 ± 0.9 for POD1; 3.3 ± 0.9 vs. 4.6 ± 0.9 for POD2; 2.2 ± 0.8 vs. 3.4 ± 1.0 for POD3; all *P* < 0.001). Analgesic pump use was significantly less frequent in the ERAS group (39.7% vs. 75.0%, *P* < 0.001). The incidence of PONV was not significantly different between groups (10.3% vs. 17.6%, *P* = 0.216) (Table 4).

**Table 4:** Comparison of postoperative pain and comfort between the two groups.

Variable	Conventional group (n = 68)	ERAS group (*n* = 68)	Statistics
VAS score, postoperative day 1 (mean ± SD)	5.8 ± 0.9	4.6 ± 1.1	t = −6.643, *P* < 0.001
VAS score, postoperative day 2 (mean ± SD)	4.6 ± 0.9	3.3 ± 0.9	t = −8.506, *P* < 0.001
VAS score, postoperative day 3 (mean ± SD)	3.4 ± 1.0	2.2 ± 0.8	t = −7.671, *P* < 0.001
Use of analgesic pump, *n* (%)	51 (75.0%)	27 (39.7%)	χ^2^ = 17.316, *P* < 0.001
PONV, *n* (%)	12 (17.6%)	7 (10.3%)	χ^2^ = 1.529, *P* = 0.216

Continuous variables were analyzed using independent-samples t tests, and categorical variables were analyzed using the χ^2^ test.

### 3.5 Postoperative complications

Overall postoperative complications were less frequent in the ERAS group than in the conventional group (27.9% vs. 61.8%, *P* < 0.001). Specifically, pulmonary infection (11.8% vs. 25.0%, *P* = 0.046), arrhythmia (2.9% vs. 11.8%, *P* = 0.049), and deep venous thrombosis (1.5% vs. 11.8%, *P* = 0.016) occurred less often in the ERAS group, whereas other individual complications did not differ significantly (Table 5). Additionally, patients in the ERAS group were more likely to have no complication (72.1% vs. 38.2%), and less likely to experience multiple complications (≥2: 2.9% vs. 16.2%, *P* = 0.020) (Table 5).

**Table 5:** Comparison of postoperative complications between the two groups.

Variable	Conventional group (*n* = 68)	ERAS group (*n* = 68)	Statistics
Any postoperative complication, *n* (%)	42 (61.8%)	19 (27.9%)	χ^2^ = 15.725 *P* < 0.001
Pulmonary infection, *n* (%)	17 (25.0%)	8 (11.8%)	χ^2^ = 3.970 *P* = 0.046
Atelectasis, *n* (%)	10 (14.7%)	5 (7.4%)	χ^2^ = 1.873 *P* = 0.171
Arrhythmia, *n* (%)	8 (11.8%)	2 (2.9%)	χ^2^ = 3.886 *P* = 0.049
Deep venous thrombosis, *n* (%)	8 (11.8%)	1 (1.5%)	χ^2^ = 5.830 *P* = 0.016
Incision infection, *n* (%)	3 (4.4%)	2 (2.9%)	χ^2^ = 0.208 *P* = 0.649
Reintubation, *n* (%)	4 (5.9%)	1 (1.5%)	χ^2^ = 1.869 *P* = 0.172
ICU readmission, *n* (%)	5 (7.4%)	3 (4.4%)	χ^2^ = 0.531 *P* = 0.466
Number of complications			
0	26 (38.2%)	49 (72.1%)	χ^2^ = 17.870 *P* < 0.001
1	31 (45.6%)	17 (25.0%)	
2	9 (13.2%)	1 (1.5%)	
3	2 (2.9%)	1 (1.5%)	

### 3.6 Nursing compliance and patient satisfaction

Nursing compliance was higher in the ERAS group for early ambulation (94.1% vs. 54.4%, *P* < 0.001) and completion of respiratory training (92.6% vs. 77.9%, *P* = 0.029). Effective sputum expectoration was numerically higher but not statistically significant (94.1% vs. 83.8%, *P* = 0.101). Patient satisfaction was higher in the ERAS group (4.4 ± 0.4 vs. 3.9 ± 0.5, *P* < 0.001) (Table 6).

**Table 6:** Comparison of nursing compliance and patient satisfaction between the two groups.

Variable	Conventional group (*n* = 68)	ERAS group (*n* = 68)	Statistics
Early ambulation compliance, *n* (%)	37 (54.4%)	64 (94.1%)	χ^2^ = 26.007, *P* < 0.001
Completion of respiratory training, *n* (%)	53 (77.9%)	63 (92.6%)	χ^2^ = 4.748, *P* = 0.029
Effective sputum expectoration, *n* (%)	57 (83.8%)	64 (94.1%)	χ^2^ = 2.698, *P* = 0.101
Nursing satisfaction (Likert 1–5, mean ± SD)	3.9 ± 0.5	4.4 ± 0.4	t = −5.369, *P* < 0.001

## 4 Discussion

This study suggests that ERAS-based refined nursing in lung cancer surgery was associated with earlier postoperative ambulation, a more rapid functional restoration, enhanced pain relief, a decreased number of complications, and a higher nursing compliance, without any negative effect on perioperative safety. These results align with current ERAS principles in lung surgery, which focus on integrated, multidisciplinary perioperative management rather than singular surgical or anesthetic modifications. The joint ERAS Society and European Society of Thoracic Surgeons (ESTS) guideline for lung surgery states clearly that early mobilization, optimized analgesia, early oral intake and early removal of tubes are excellent strategies to enhance outcomes following lung resection (8).

Early mobilization is a cornerstone of thoracic ERAS pathways. ERAS lung surgery guideline specifically recommends mobilization on the day of surgery or within 24 h, because immobility deteriorates pulmonary function and predisposes patients to thromboembolism (8). Recent clinical data lend additional support to this recommendation: an early-ambulation study after lung resection found that greater early walking distance was correlated with fewer postoperative pulmonary complications and a shorter hospital stay (12). One study also suggests patients who ambulated on the day of surgery have better outcomes and less complications than those patients who remain in bed (11). In our series, ERAS-based refined nursing practice appeared to translate these principles into routine clinical practice: the ERAS patients had an earlier ambulation, walked further within the initial 48 h, and attained an enhanced functional mobility score. Rather than being a purely protocol-level change, this reflects a tangible improvement in early physical function, which is known to be strongly linked to subsequent clinical outcomes (9).

Analgesia is a major factor to allow early methodical mobilization. Thoracic surgery is very painful, and suboptimal analgesia is a barrier to deep breathing, coughing, and ambulation (22). Thoracic anesthesia and analgesia in the ERAS context applies attention principle and highly recommends the use of multimodal opioid-sparing approaches to achieve adequate analgesia with minimal sedation and gastrointestinal side effects (23). A recent study on VATS demonstrated that a multimodal pre-emptive analgesia regimen could reduce postoperative pain and opioid consumption, accelerating the recovery (24). The lower pain levels during the first 3 days after surgery, combined with less use of patient-controlled analgesia, in our ERAS group suggests that the experienced nursing staff successfully applied multimodal analgesia in daily routine in routine practice. This probably led to not only better comfort but also early and willing mobilization for the patients as well as their participation in respiratory training.

The rapid postoperative recovery reported in this study is consistent with the present ERAS results. Several systematic reviews and meta-analyses in various surgical specialties, including thoracic surgery, have demonstrated that ERAS protocols reduce post-operative LOS and complications (25). Meta-analyses concentrated on lung cancer have shown that ERAS protocols significantly lowered complication rates and length of hospital stay without elevating readmission or mortality (26). Even more recently, a systematic review of thoracic ERAS protocols demonstrated these are associated with a consistent reduction in LOS and frequently with a decrease in pulmonary and cardiac complications following lung resection (5). In parallel, observational studies of thoracic ERAS implementation also describe shorter LOS and decreased ICU admission rates post-lung resection with the adoption of standardized protocols (27, 28). Therefore, our observation that ERAS-guided refined nursing care led to earlier bowel function recovery, shorter duration of chest tubes and urinary catheters, and abbreviated hospitalization is in strong agreement with the findings above, and consolidates that nursing delivery is a critical factor in the success of ERAS on the bedside.

The complication profiles in our study are also indicative of a potential protective association of ERAS-based refined nursing. A pooled analysis examining 21 different ERAS studies demonstrated that ERAS protocols reduce the risk of postoperative complications by about a third in comparison to conventional care (21, 25). In thoracic surgery in particular, ERAS protocols have been linked to reductions in overall complications, pulmonary complications, and hospital costs (1, 29). In our series, the ERAS group had fewer complications overall, and the differences were particularly striking with respect to pulmonary infection, DVT, and arrhythmia, which are closely associated with immobility, impaired ventilation, and increased stress response. These findings are in line with studies that report that ultra-early rehabilitation after thoracic surgery is a viable, safe, and outcome-enhancing approach (30). Taken together, both the current literature and our findings indicate that specialized nursing care provided according to ERAS protocols has the potential to alter multiple modifiable risk factors at once and so to shift the overall complication profile toward a more favorable distribution.

A key strength of this study is the direct assessment of nursing adherence and patient outcomes. Many ERAS articles emphasize traditional surgical end points, such as LOS and rate of complications; fewer offer a granular analysis of compliance with early ambulation, respiratory training, or patient satisfaction. Recent studies of postoperative mobility have demonstrated that objective measures of activity, including step counts, distance walked, or standardized functional tests, are associated with recovery and postoperative complications, 1–4 yet these parameters are rarely monitored in routine clinical care (10). The results that ERAS-based refined nursing significantly increased compliance to early mobilization and respiratory exercises and at the same time refined nursing satisfaction scores indicate that the nursing staff is integral to converting ERAS guidelines into patient conduct and perceived quality of care. This is in line with wider ERAS literature which underscores the paramount importance of multidisciplinary commitment and patient education rather than protocols alone in contributing to pathway success (14, 31).

In summary, our findings support that ERAS-based refined nursing constitutes the core operational component of thoracic ERAS pathways: at the bedside, nurses simultaneously manage analgesia, mobilization, respiratory training, early feeding, and tube management to amalgamate multiple evidence-based components into one recovery path. The synergistic approach is probably essential in lung cancer surgery in particular because most such patients encountered are old and have comorbidities and no robust pulmonary reserve.

There are some limitations that should be considered. First, this was a retrospective, single-center study, which may introduce selection bias and limit generalizability. Second, because group allocation was non-randomized, residual confounding from unmeasured factors cannot be excluded; therefore, causal inference should be made cautiously. Third, we focused on early postoperative outcomes during hospitalization and did not assess long-term functional status, quality of life, or oncologic outcomes. Future multicenter prospective studies with longer follow-up are warranted to confirm these findings and evaluate cost-effectiveness.

## 5 Conclusion

ERAS-based refined nursing was found to be associated with earlier postoperative ambulation, faster recovery, better pain control, a lower incidence of postoperative complications, and higher nursing compliance and patient satisfaction in patients undergoing lung cancer surgery. These findings highlight the important role of ERAS implementation into perioperative care. ERAS-guided refined nursing appears to be a safe and effective perioperative management strategy but warrants further validation in prospective and multicenter studies.

## Data Availability

The original contributions presented in the study are included in the article/Supplementary Material, further inquiries can be directed to the corresponding author.
